# A Rare Case of Anemia Secondary to Lice Infestation

**DOI:** 10.7759/cureus.27057

**Published:** 2022-07-20

**Authors:** Justin Slovin, Bilal A Niazi, Mayuri Kinkhabwala, Alexandria Ang, Syed Sirajuddin

**Affiliations:** 1 Internal Medicine, Hackensack University Medical Center School of Medicine, Hackensack, USA; 2 Internal Medicine, Hackensack Meridian Health - Palisades Medical Center, North Bergen, USA; 3 Family Medicine, Hackensack Meridian Health - Palisades Medical Center, North Bergen, USA

**Keywords:** ivermectin, hygiene, homeless, anemia, lice

## Abstract

Lice are parasitic infections that can infect humans and survive by consuming host blood. They are most commonly associated with a lack of hygiene and occur most commonly in school-age children, homeless populations, and densely populated areas. Lice infections can potentially lead to vector-containing illness and are rarely complicated by acute blood loss anemia. These side effects, while exceedingly rare, are often very significant and potentially life-threatening. Here we present a rare case of severe anemia found in a patient with chronic lice colonization. We hope our findings will broaden the differential for acute anemias and raise awareness of lice infection as a possible cause of acute anemia.

## Introduction

Lice are a diverse group of parasitic insects that subsist via the consumption of the blood of mammals. From this group, only two species are known to primarily colonize humans, *Pediculus humanus* and *Phthirus pubis* [1]. In most hosts, they cause only minor itching, but in rare cases, they can cause more severe sequelae, including anemia and transmission of infectious diseases such as typhus, trench fever, and relapsing fever [2,3]. Anemia in these patients occurs primarily due to prolonged consumption of blood by the lice in the setting of chronic infection [4]. Worldwide, the most common causes of anemia include blood loss, lack of dietary iron, pregnancy, infectious disease, and malabsorption [5-7]. Among these causes, iron deficiency anemia is the most common cause of anemia among humans, with common symptoms including fatigue, weakness, restless legs, nail changes (koilonychia), pica, and cold intolerance [6,8,9]. This case report will bring awareness to the more severe side effects of lice infestation, specifically iron deficiency anemia.

## Case presentation

The patient is a 44-year-old female who is homeless with no significant past medical history and presented to the emergency department after being found incapacitated in the street. During the initial encounter, the patient reported feeling tired and waking up in the hospital. She denied any history of bleeding. She had no known medical history and did not take any medications or herbal supplements. She reported regular menses every 28 days, lasting 5-6 days, without any excessive bleeding. She reported being homeless for one year and denied any tobacco, alcohol, or recreational drug use.

On initial evaluation, vital signs were significant for blood pressure 128/84 mmHg, heart rate of 72 beats per minute, afebrile, and oxygen saturation of 100% on room air. On physical examination, the general appearance revealed unkempt hygiene, pale skin, and widespread lice infestation. She was found to have pale conjunctiva, and her cardiac exam was remarkable for a 1/6 systolic murmur at the left second intercostal space but otherwise unremarkable. Laboratory results were remarkable for hemoglobin (Hgb) 2.8 g/dL, hematocrit 9.9%, mean corpuscular volume (MCV) of 59.3 fL, total iron of <10 ug/dL, transferrin of 2%, ferritin 10 ng/mL, and reticulocyte count 0.5%. Additionally, she was found to have lactate dehydrogenase 205 U/L, haptoglobin 106 mg/dL, total bilirubin 0.6 mg/dL, and direct bilirubin 0.1 mg/dL. A review of records revealed a previous Hgb of 14.3 g/dL, with an MCV of 86 fL about one year ago. Evaluations for prothrombin time (PT), partial thromboplastin time (PTT), white blood cell (WBC) count, platelet count, bilirubin levels, blood urea nitrogen, and creatinine were within normal limits. B12 was found to be 670 and folate 12.0 ng/mL. Blood alcohol levels were less than 10 mg/dL. Her urine toxicology report was unremarkable. Titers for cytomegalovirus, malaria, babesia, anaplasmosis, ehrlichiosis, and Lyme disease were unremarkable. Her stool was negative for occult blood. EKG revealed normal sinus rhythm, with a rate of 92 bpm. The patient could not get a CT scan due to her lice infestation. She was given a treatment of permethrin cream and lindane shampoo and was administered three units of packed red blood cells (RBC) and continuous crystalloid resuscitation. 

Three units of packed RBC were administered and repeat laboratory values revealed improvement to Hgb of 9 g/dL, hematocrit to 28.5%, and MCV 78.7 fL. The trend of Hgb is displayed in Table 1.

**Table 1:** Hemoglobin levels during hospital course This table displays the trend in hemoglobin levels. Three units of packed red blood cells were delivered on hospital day 1 when the hemoglobin level was found to be low. There was an appropriate response in the hemoglobin level the following day. The hemoglobin levels were stable subsequently through the rest of the hospital course without requiring any additional transfusions.

Hospital Day	Hemoglobin Level (g/dL)
1	2.8
2	9.0
3	8.5
4	9.2
5	9.0
6	9.1

She was treated with a dose of oral ivermectin after the permethrin and lindane were ineffective in eradicating the lice. Additionally, the patient consented to head shaving to assist in this eradication. Her itchiness ultimately resolved, and her Hgb remained stable through her hospital course. Social work assisted in finding placement at a homeless shelter and scheduling appropriate medical follow-ups. The patient was ultimately discharged after a six-day hospital course. 

## Discussion

Lice infestation can quickly become an acute condition, with female lice laying approximately eight eggs per day, totaling hundreds in their lifetimes overall. These parasitic insects prosper in hot and humid environments and are dependent on a host organism, usually dying within 1-2 days of being removed. Given the lack of access to proper hygiene and the commonly hot and humid living conditions, the homeless population is particularly at risk for lice colonization [10]. Our patient presented in unkempt attire and reported to have been homeless for over one year, consistent with a high-risk setting for lice infection.

The most common symptom of lice infestation is pruritus at the location of bites, secondary to immunoglobulin E (IgE)-mediated allergic reaction to lice saliva in the skin [11,12]. Additionally, because lice feed primarily on host blood, chronic infection can lead to anemia. However, it is important to rule out other causes of anemia such as acute blood loss, nutritional deficiencies, malabsorption, and even heavy menstrual bleeding in women [6]. The diagnosis of lice infection will typically involve a physical examination visualizing lice in the hair and on the skin. The lice can be seen with the naked eye or with the assistance of a magnifying glass and comb. Additionally, lice nits can be visualized with the assistance of a Wood’s lamp [13]. In our specific case, the lice were able to be visualized without any assistance with a comb or magnifying glass due to their large number. Additionally, the patient’s iron deficiency anemia was first suspected when laboratory evaluation revealed Hgb 2.6 g/dL, with a microcytic MCV, and severely low iron and ferritin. However, other causes of microcytic anemia, such as acute bleeding, nutritional deficiencies, and infections, needed to be ruled out. We screened out heavy menstrual bleeding in history gathering, as the patient reported regular menses without excessive bleeding. Additionally, further evaluations revealed negative urine toxicology, negative stool occult blood, normal platelet and WBC counts, normal B9 and B12 levels, normal PT and PTT, normal total bilirubin and direct bilirubin, and normal infectious titers including those for malaria, babesia, and Lyme disease. Given that our patient presented with a microcytic anemia with low iron and ferritin levels in the setting of generalized lice infection, and that the other causes of microcytic anemia had been ruled out, the etiology of iron deficiency anemia was ultimately found to be due to chronic host consumption of blood from chronic lice infection.

Treatments for lice can depend on the severity of the infestation. Mild colonization with lice can be treated topically with permethrin cream, while more severe colonizations or those refractory to topicals can involve oral treatments with oral ivermectin [14]. Our patient was treated with permethrin and lindane topicals, as well as a course of oral ivermectin. She was found to have symptomatic improvement of her itchiness symptoms and her Hgb levels were stable after transfusion. Per our research, this is a rare and serious issue that we would like to bring awareness to. Lice are an important issue to consider when caring for high-risk populations including school-age children, as well as homeless adults or individuals with poor hygiene. Suspicion should be higher if these patients present with symptoms of pruritus, fatigue, or neurological symptoms. Treatment is very important due to the high rate of transmission from one infected individual to close contacts.

## Conclusions

Lice can colonize humans, especially children and those with poor hygiene such as the homeless and those who live in heavily populated areas, and survive by consuming their blood. Colonization with lice can potentially lead to vector-containing illness as well as acute blood loss anemia. These side effects, while exceedingly rare, are often very significant and potentially life-threatening. This report showcased a rare case of severe anemia found in a patient with chronic lice colonization. We hope our findings will broaden the differential for acute anemias and raise awareness of lice infection as a possible cause of acute anemia.
